# The Super Brains app: a psycho-educative program for adults with ADHD

**DOI:** 10.1192/j.eurpsy.2023.73

**Published:** 2023-07-19

**Authors:** J. S. Kooij

**Affiliations:** ^1^Psychiatry, AUMC/VUMc, Amsterdam; ^2^Adult ADHD, PsyQ, The Hague, Netherlands

## Abstract

**Abstract:**

Digital treatment for neurodevelopmental disorders is being developed in order to treat patients online when possible, to reduce waiting lists and to improve efficacy and efficiency of treatment. In this presentation, experiences with the so called Start Program of the Super Brains app for adults with ADHD are presented. The Super Brains app has been developed by Rutger den Hollander, who himself has ADHD and owns an ICT company, together with the speaker of this presentation and Parnassia Groep in the Netherlands.

The Start Program is part of the Super Brains app, and meant for patients referred for treatment, who have to wait on often long waiting lists. Now they have no longer to wait, but can start immediately by preparing for diagnostic assessment by filling in questionnaires, and with psycho-education, lifestyle tips and support by experience experts, who welcome them in the app and show them around.

First data on the use of different parts of the Start Program, the activity of the patients in the app and the satisfaction of patients will be presented. We also aim to study whether the Start Program is effective in reducing severity of ADHD symptoms during the waiting time. Super Brains can be adjusted for use in other (neurodevelopmental) disorders easily (https://www.superbrains.nl/?lang=en).

**Disclosure of Interest:**

None Declared

